# *Staphylococcus aureus* and Coagulase-Negative Staphylococci from Bloodstream Infections: Frequency of Occurrence and Antimicrobial Resistance, 2018–2021

**DOI:** 10.3390/life13061356

**Published:** 2023-06-09

**Authors:** Nicola Serra, Paola Di Carlo, Maria Andriolo, Giovanni Mazzola, Elena Diprima, Teresa Rea, Antonio Anastasia, Teresa Maria Assunta Fasciana, Luca Pipitò, Giuseppina Capra, Anna Giammanco, Antonio Cascio

**Affiliations:** 1 Department of Public Health, University Federico II of Naples, 80131 Napoli, Italy; 2 Department of Health Promotion, Maternal-Childhood, Internal Medicine of Excellence “G. D’Alessandro”, Infectious Disease Unit, University of Palermo, 90127 Palermo, Italy; 3 Clinical Pathology Unit, S. Elia Hospital, 93100 Caltanissetta, Italy; 4 Infectious Disease Unit, Provincial Health Authority of Caltanissetta, 93100 Caltanissetta, Italy; 5 Hypatia Degree Course, Caltanissetta, University of Palermo, 90127 Palermo, Italy; 6 Department of Health Promotion, Maternal-Childhood, Internal Medicine of Excellence “G. D’Alessandro”, University of Palermo, 90127 Palermo, Italy; 7 Department of Health Promotion, Maternal-Childhood, Internal Medicine of Excellence “G. D’Alessandro”, Microbiology and Virology Unit, University of Palermo, 90127 Palermo, Italy

**Keywords:** *Staphylococcus aureus*, coagulase-negative staphylococci, antimicrobial resistance, SARS-CoV-2

## Abstract

Background: The abuse of antibiotics during the SARS-CoV-2 pandemic might have disrupted efforts to curb the further development and spread of the antimicrobial resistance of *Staphylococcus aureus* infection and *Staphylococcus* spp. coagulase-negative (CoNS) agents of nosocomial bloodstream infections (NBSIs). The purpose of our work was to study the resistance patterns of *Staphylococcus aureus* and CoNS through the analysis of blood cultures in hospitalized SARS-CoV-2-positive and SARS-CoV-2-negative patients (pts.). Materials and methods: During the period January 2018–June 2021, a retrospective case–control study was performed on blood cultures positive for *Staphylococcus* spp. detected in 177 adult pts. (≥18 years old) hospitalized for >48 hours at Sant’Elia Hospital, Caltanissetta. Results: *Staphylococcus aureus* was isolated in 33.9% of blood culture samples, and among CoNS, the most frequent strains were *Staphylococcus capitis* (18.6%) and *Staphylococcus hominis* (18.1%). Patients aged ≥ 65 years, with a greater number of males, comprised the SARS-CoV-2-negative pts. (71.8% vs. 52.2%, *p* = 0.0154). Among the SARS-CoV-2-positive patients, the significant resistance of *Staphylococcus aureus* was only observed for erythromycin (57.1%). The oxacillin resistance of *Staphylococcus capitis* was higher in SARS-CoV-2-positive than in negative pts. (90% and 78.3%, respectively). Comparing the two groups, we found an increase in resistance in SARS-CoV-2-negative patients for the following antibiotics: gentamicin for *Staphylococcus aureus* (*p* = 0.007), clindamycin and erythromycin (*p* = 0.012) for *Staphylococcus hominis* and oxacillin and rifampicin for *Staphylococcus haemoliticus* (*p* = 0.012). Conclusions: Our study confirms the relevance of oxacillin-resistant *Staphylococcus aureus* in being responsible for bloodstream infection and draws attention to highly oxacillin-resistant CoNS such as *Staphylococcus capitis*. The presence of resistant strains of CoNS in hospitals can be worrying, as it limits treatment options and worsens outcomes. The Infection Control Committee (ICC) recommends new treatment strategies to decrease colonization and infections. As part of the implementation of a bloodstream infection prevention program, the authors encourage the introduction of a report on the antimicrobial resistance of hospital bacteremia due to CoNS.

## 1. Introduction

The severe acute respiratory syndrome coronavirus 2 (SARS-CoV-2)-related infections (COVID-19) pandemic has exposed both old and new issues in healthcare-associated infections, especially bloodstream infections [1,2]. Moreover, the abuse of antimicrobials during the SARS-CoV-2 pandemic has exacerbated the problem of antimicrobial resistance [3,4]. The change in antibiotic prescribing practices in hospitalized and outpatient settings, emergency department crowding and hospital transformations due to the COVID-19 pandemic have influenced both empiric antibiotic treatment in patients with severe febrile illness and the utilization of blood cultures in the diagnosis of suspected cases of bacteremia [5]. Despite advancements in microbiologic diagnostic techniques, blood cultures (BCs) remain a first-line tool for diagnosing bloodstream infections. Their diagnostic value may be affected when a microorganism is a common skin commensal [6]. Due to patient- and procedure-related changes, coagulase-negative staphylococci (CoNS) are now emerging nosocomial pathogens with a significant disease spectrum and an extensive antimicrobial resistance profile [7,8,9]. During and after the pandemic’s waves, the epidemiology of microorganisms responsible for hospital-acquired infections showed changing patterns [2,5,7,9,10]. According to the WHO’s regions, *Staphylococcus* aureus infection was the most common Gram-positive microorganism responsible for hospital-acquired infections (HAIs) in the EMRO region, and coagulase-negative staphylococci was the most common microorganism in the WPRO and EURO regions [11,12].

CoNS are able to form biofilms to enhance their virulence, as well as to protect themselves from the diffusion of antibiotics into the host cells. Many CoNS have become resistant to methicillin, and there is evidence that they are reservoirs of the SCCmec complex; therefore, advanced phenotypic and genotypic studies (particularly on SCCmec properties) are crucial for their further characterization and better understanding. Recently, the presence and regulation of virulence factors typical for S. aureus in CoNS strains and regulatory genes attributed to *S. aureus* in CoNS isolates indicated that the cognition of the prevalence and regulation of virulence factors, as well as the antibiotic resistance of CoNS isolates, are important for the better control and treatment of CoNS infections [13,14]. Excess skin bacteria yielded from blood samples are over-represented in acute care settings, such as CoNS and *Corynebacterium* species (*Corynebacterium striatum*), compared to previous isolates, suggesting the need to identify contaminant cultures early to avoid antibiotic treatment [6,13,14]. Therefore, the fundamental role of bacteria identified in a nosocomial setting is under debate, especially when the bacterium identified in blood cultures is *Staphylococcus* spp. coagulase-negative (CoNS) [9,15,16].

A surveillance study showed an increase in the antimicrobial resistance of CoNS in hospitalized Sicilian patients [17]. Therefore, to monitor the evolution of CoNS resistance, we performed the retrospective surveillance of the resistance patterns of *Staphylococcus* spp. through the analysis of blood cultures in SARS-CoV-2-positive and negative patients admitted at Sant’Elia Hospital, Caltanissetta, Central Sicily.

## 2. Materials and Methods

This study is a retrospective comparative investigation of blood culture (BC) samples positive for *Staphylococcus* spp. in adult (≥18 years old) SARS-CoV-2-positive and negative patients hospitalized for at least 48 hours at Sant’Elia Hospital in Caltanissetta from January 2018 to June 2021. 

The samples for the blood cultures were collected aseptically via peripheral venipuncture from patients with a suspected bloodstream infection according to CDC guidelines, as previously reported [18,19].

Each positive BC result was assessed for contamination (i.e., false positive), and according to the Blood Specimen Collection of the National Healthcare Safety Network [20], the false-positive isolates were excluded from the study [6].

Patients’ records included age, sex, isolated bacteria, hospital wards and antimicrobial susceptibility patterns. The data records were obtained from a database using institutional electronic microbiological information. Bacterial identification and antimicrobial susceptibility testing for *Staphylococcus aureus* and CoNS were carried out using the Vitek-2 System (Bio-Mérieux, Marcy l’Etoile, France) at the Sant’Elia Clinical Pathology and Microbiology Unit, as previously reported. If necessary, the Phoenix Automated Microbiology System (Becton Dickinson Diagnostic Systems, Sparks, United States) was also available. A routine surveillance measure for infection control was applied at Sant’Elia Hospital, Caltanissetta, Italy, as part of the GISIO and SPIN UTI Italian surveillance projects and the Sicilian antimicrobial resistance surveillance system [21]. According to the European Committee on Antimicrobial Susceptibility Testing (EUCAST) for *Staphylococcus aureus* and CoNS breakpoints [22,23], the antimicrobial susceptibility test for the strains was determined as reported above [18,21,24].

### Statistical Analysis

Data are presented as numbers and percentages for categorical variables, and the continuous data are expressed as the mean ± standard deviation (SD) unless otherwise specified.

Chi-square and Fisher’s exact tests were performed to evaluate significant differences in proportions or percentages between the two groups. Mainly, Fisher’s exact test was used where the chi-square test was not appropriate.

The multiple-comparison chi-square tests defined significant differences among percentages for unpaired data. If the chi-square test was significant (*p*-value < 0.05), a residual analysis with the Z-test was performed. In addition, the chi-square goodness of fit was used to evaluate significant differences among three or more modalities of a variable.

In the case of paired data, the multiple-comparison Cochran’s Q-test was used to compare the differences among percentages under consideration of the null hypothesis that there were no differences among the variables. When the Cochran’s Q-test was positive (*p*-value < 0.05), a minimum required difference for a significant difference between two proportions was calculated using the minimum required differences method with the Bonferroni *p*-value corrected for multiple comparisons.

Tests for normal distribution were performed using the Shapiro–Wilk test. The t-test was used to test the differences between two means of unpaired data. An alternative nonparametric test, such as the Mann–Whitney test, was used to compare two independent samples when the distributions were not normal. 

We considered all statistical tests with a *p*-value < 0.05 to be significant. All data were analyzed using MATLAB’s statistical toolbox, version 2008 (MathWorks, Natick, MA, USA) for 32 bit Windows.

## 3. Results

In Table 1, we report the demographic characteristics of the patients, including the *Staphylococcus* species distribution isolated in the blood cultures, patients with coronavirus disease, length of hospital stay, patients admitted to intensive care settings (ICUs) and mortality rate. The duration of hospital stay was 36.5 ± 35.5 days in our studied population. Most were patients aged 65 years and older, with a prevalence of the male gender (67.7%). In addition, Table 1 shows our sample stratified into SARS-CoV-2-negative patients and SARS-CoV-2-positive patients. Finally, in the last column, a comparison between the SARS-CoV-2-negative and positive groups for each parameter considered is shown, including all staphylococci detected. 

As shown in Table 1, among *Staphylococcus* species in SARS-CoV-2-negative patients, there was a significantly higher frequency of *Staphylococcus aureus* (35.1%, *p* < 0.0001)*, Staphylococcus capitis* (17.6%, *p* = 0.0005) and *Staphylococcus hominis* (17.6%, *p* = 0.0005). Similarly, in SARS-CoV-2-positive patients, it was *Staphylococcus aureus* (30.4%, *p* < 0.0001), *Staphylococcus capitis* (21.7%, *p* = 0.0038) and *Staphylococcus hominis* (19.7%, *p* = 0.0171). In addition, by comparing the SARS-CoV-2-negative and positive groups (last column in Table 1), we observed a greater number of males who were SARS-CoV-2-negative (71.8% vs. 52.2%, *p* = 0.0154). Regarding the mortality rate, we found a significantly higher mortality percentage in the SARS-CoV-2-positive patients (41.3% vs. 29.8%, *p* = 0.0119).

In Table 2, we report the percentages of antimicrobial resistance for each *Staphylococcus* spp. isolated in the group negative for SARS-CoV-2.

Notably, for *Staphylococcus aureus,* the antibiotics penicillin (93%, *p* < 0.05), erythromycin (60%, *p* < 0.05), levofloxacin (62.2%, *p* < 0.05) and oxacillin (65.2%, *p* < 0.05) were the antimicrobials with the most resistance. For *Staphylococcus capitis,* the antibiotics gentamicin (73.9%, *p* < 0.05), levofloxacin (69.6%, *p* < 0.05) and oxacillin (78.3%, *p* < 0.05) were the antimicrobials with the most resistance. For *Staphylococcus epidermidis,* oxacillin (85.7%, *p* < 0.05) was the antibiotic with the most resistance. Finally, for *Staphylococcus hominis,* clindamycin (77.3%, *p* < 0.05), erythromycin (90.9%, *p* < 0.05) and oxacillin (76.2%, *p* < 0.05) were the antibiotics with the most resistance.

Table 3 shows the percentages of antibiotic resistance for each *Staphylococcus* spp. isolate in SARS-CoV-2-positive patients.

From Table 3, we can observe that among all of the antibiotics used, *Staphylococcus aureus* showed a significantly higher resistance to penicillin (85.7%, *p* < 0.05) and erythromycin (57.1%, *p* < 0.05) in comparison to the others. *Staphylococcus capitis* showed the higher resistance to gentamicin (80%, *p* < 0.05), levofloxacin (80%, *p* < 0.05) and oxacillin (90%, *p* < 0.05). 

*Staphylococcus epidermidis* showed higher resistance to erythromycin (100%, *p* < 0.05) and oxacillin (100%, *p* < 0.05). Finally, for *Staphylococcus hominis*, levofloxacin and oxacillin were the antibiotics with the most resistance among all antimicrobials used (55.6%, *p* < 0.05 for both). 

In Table 4, we report the comparison of the antibiotic resistance of *Staphylococcus* spp. in SARS-CoV-2-infected and noninfected groups. 

From Table 4, we can find greater resistance to antimicrobials in the *SARS-CoV-2-*negative group than the SARS-CoV-2-positive group for gentamicin (*Staphylococcus aureus*: 35.6% vs. 0.0%, *p* = 0.007), clindamycin (*Staphylococcus hominis*: 77.3% vs. 22.2%, *p* = 0.012), erythromycin (*Staphylococcus hominis*: 90.9% vs. 44.4%, *p* = 0.012), oxacillin (*Staphylococcus haemolyticus*: 100% vs. 40%, *p* = 0.012) and rifampicin (*Staphylococcus haemolyticus*: 84.6% vs. 20%, *p* = 0.022).

## 4. Discussion

In patients presenting with severe febrile illness, the current diagnostic modalities cannot sufficiently distinguish between bacterial and viral disease during the early stages of a diagnostic workup. In the case of suspected sepsis, blood cultures (BCs) remain the gold-standard test. In hospital settings, comorbidities, previous medication at home, and the placement of lines can induce the suspicion of *bacterial* contamination and subsequent infections. The challenges experienced by public health systems, such as hospitals in the Italian healthcare system, include staffing shortages, high hospitalization rates and supply constraints, which may have led to differences according to the circumstances of each facility, changes in infection control practices and antibiotic overuse [25]. Consequently, the number of positive blood cultures per patient per day may be increased, reflecting both the increase in secondary bacterial infections and the chance of contamination [25,26]. Our study focused on bacteremia due to *Staphylococcus* spp. in patients hospitalized in a single center in southern Italy during the first and second waves of the COVID-19 pandemic to study the trends in Gram-positive microorganisms, such as *Staphylococcus* spp. coagulase-positive and negative causative agents, in bloodstream infections. Our results show a significantly greater frequency of bloodstream infections due to *Staphylococcus aureus* in approximately one-third of the patients, which is known to be a pathogen and not a contaminant, followed by *Staphylococcus capitis* and *Staphylococcus hominis* in both groups (positive and negative for SARS-CoV-2). A significant difference in the distributions of *Staphylococcus aureus* and CoNS was not observed (*p* = 0.80).

In the SARS-CoV-2-negative group, *Staphylococcus aureus* showed resistance to penicillin > 80%, as known since 1960, in both groups [27]. In contrast, the rate of resistance to oxacillin was significantly high (65.2%) in the SARS-CoV-2-negative group, as reported by other studies that showed the tendency of MRSA to increase during the COVID-19 era in both positive and negative subjects due to the initial abuse of antimicrobials [28,29,30]. This trend of antibiotic resistance for *Staphylococcus aureus* explains the significant level of resistance to erythromycin (60%) and levofloxacin (62.2%) in the SARS-CoV-2-negative group. In contrast, in the positive SARS-CoV-2 group, we only found a significant resistance to erythromycin (57.1%) [31,32]. The lack of significance in the SARS-CoV-2-positive group could be due to the uncommon use of levofloxacin in hospitalized patients for staphylococcal infections, because it is known that levofloxacin combined with the standard treatment for S. aureus bacteremia neither decreases mortality nor the incidence of serious diseases. Conversely, macrolide resistance in staphylococci is common and associated with the presence of many molecular determinants and correlates with resistance to methicillin [33,34]. We reported a higher mortality rate in SARS-CoV-2-positive patients than in negative hospitalized patients (41.3% vs. 29.8%; *p* = 0.0119). This result strengthens the previous hypothesis that coagulase-negative staphylococci infections are an emerging problem as pathogens causing secondary bacteremia infection in patients with moderate and severe pneumoniae caused by viruses [35,36].

Among CoNS, our data showed a prevalence of blood cultures positive for *Staphylococcus capitis* and *Staphylococcus hominis* in SARS-CoV-2-negative and positive patients. *Staphylococcus capitis* has been associated with bloodstream infection in neonatal intensive care units [37] and rarely in hospitalized adult patients [38]. Notably, this study observed a high percentage of oxacillin resistance in S. capitis in the SARS-CoV-2-positive and negative groups (90% and 78.3%, respectively). Moreover, *Staphylococcus capitis* showed antibiotic resistance to gentamicin and levofloxacin in both groups. *Staphylococcus capitis* is responsible for hospital-acquired infection, and a thorough investigation should be conducted to identify the control of the hospital cluster [39].

The oxacillin resistance of *Staphylococcus hominis* was observed in both groups, but there was a higher percentage in the SARS-CoV-2-negative than positive group (76.2% versus 55.6%); moreover, in the COVID-19-negative group, the resistance to erythromycin and clindamycin was more significant than other antimicrobials.

Erythromycin and clindamycin resistance in SARS-CoV-2-negative patients is because of high macrolide class abuse, geographical distribution in Italy and the complexity of the resistance phenotypes with cross-resistance to lincomycin and erythromycin [40]. Moreover, a possible explanation for the high rate of *Staphylococcus aureus* oxacillin resistance found in our study and, in general, in CoNS should be a consequence of these Gram-positive microorganisms sharing the same niches of colonization with *Staphylococcus aureus*, allowing for the horizontal gene transfer (HGT) of several genes and mobile elements encoding for antimicrobial resistance [7,8,14,41].

A comparative statistical analysis of the two groups showed increased resistance in SARS-CoV-2-negative patients for the following antimicrobials: gentamicin for *Staphylococcus aureus*, clindamycin and erythromycin for *Staphylococcus hominis* and oxacillin and rifampicin for *Staphylococcus haemoliticus*. Except for oxacillin administered intravenously during hospitalization, the following antimicrobials are available in the Italian national health system by general practitioner and pediatric prescription: gentamicin, rifampicin, clindamycin and erythromycin.

In 2020, the Italian Medicines Agency report showed that approximately 90% of antibiotic consumption reimbursed by the National Health System is prescribed by general practitioners (GPs) and pediatricians and dispensed by the pharmacies of the Italian territory [42].

Critical articles have focused on the role of macrolides in mitigating the viral load of SARS-CoV-2 in some patients, forgetting the harmful effects of antimicrobial resistance caused by selective pressure on individuals’ natural microbiota and the phenomenon of antimicrobial resistance [43].

Despite guidelines and programs by government agencies to enhance the quality of antibacterial prescriptions and reduce the rate of antibiotic abuse, it remains approximately fixed, mainly among vulnerable populations, such as the elderly [44].

According to the age distribution of the Italian people, which is the European country with the highest number of elderly subjects (≥65 years), the mean age of our sample was ≥65 years [45].

In addition, one of the leading causes of antimicrobial resistance is the misuse of antibiotics in terms of self-prescriptions, incomplete therapies, and missing doses. Especially during the COVID-19 pandemic and lockdown period, patients, even if negative for COVID-19 but with febrile symptoms, bought antibiotics at the pharmacy without a medical prescription, as reported in studies from England [46,47]. The family doctor is important and is a reference point for subjects, especially the elderly and children. These findings are similar to those of a study conducted in Greece and Italy, which pointed out that 10% of parents would consider giving their children antibiotics without previous medical advice and that 44% received antibiotic recommendations from their family doctor over the phone [48,49]. In brief, despite coagulase-negative staphylococci being responsible for nosocomial catheter-related bloodstream infections, especially in immunocompromised populations, no international or local surveillance systems are available, and data regarding their resistance patterns were collected from the literature. As part of implementing bloodstream infection prevention programs, the authors encourage the introduction of a regional report on the antimicrobial resistance of hospital bacteremia due to coagulase-negative *staphylococcus*.

In conclusion, bloodstream infections (BSIs) caused by CoNS are one of the most prevalent nosocomial infections among all age groups. Coagulase-negative staphylococci (CoNS) are significant in causing illness and contributing to healthcare costs, especially in the elderly with significant comorbidities.

Furthermore, resistant strains of CoNS can limit treatment options and worsen outcomes. To prevent colonization and reduce infections, advanced research must understand CoNS as a reservoir for resistance and virulence genes and develop innovative treatment strategies, according to the Infection Control Committee (ICC).

## 5. Limitations

Our study was conducted under strict regulations for antibiotics and antimicrobial stewardship at S’Elia Caltanissetta Hospital. These regulations vary by setting, so it is important to take this into account when considering our findings. Unfortunately, we could not determine the effect of inpatient antibiotic administration on the outcome of BCs drawn in all units, including the ICU. Additionally, we performed some statistical analyses on a small sample of data, which may increase the probability of statistical bias. To reduce this bias, we used a statistical test for small samples or continuity corrections. Moving forward, we plan to conduct a multicenter study with a larger sample size to confirm our preliminary results.

## Figures and Tables

**Table 1:** Patient characteristics and staphylococcal species isolated in the blood of SARS-CoV-2-negative and positive groups.

Parameters	Total Sample	SARS-CoV-2- Negative	SARS-CoV-2- Positive	Negative vs. Positive *p*-Value (Test)
*Patients*	177	131	46	
*Age*				
Mean ± SD	65.5 ± 16.6	66.1 ± 16.3	66.7 ± 17.4	
Median (IQR)	69 (55, 78)	69 (55, 77)	71 (57, 82)	0.42 (MW)
*Gender*				
Male	66.7% (118)	71.8% (94)	52.2% (24)	
Female	33.3% (59)	28.2% (37)	47.8% (22)	0.0154 * (C)
*Hospital length of stay (days)*				
Mean ± SD	36.5 ± 35.5	36.7 ± 37.9	35.7 ± 28.1	
Median (IQR)	22 (13, 43.25)	22 (13, 43)	27 (14, 48)	0.57 (MW)
*Mortality percentage*	32.8% (58)	29.8% (39)	41.3% (19)	0.0119 * (C)
*Operative unit*				0.23 (C)
Non-ICU	57.6% (102)	55.0% (72)	65.2% (30)	
ICU	42.4% (75)	45.0% (59)	34.8% (16)	
*Staphylococcus aureus*	33.9% (60) *	35.1% (46) *	30.4% (14) *	
*Staphylococcus capitis*	18.6% (33) *	17.6% (23) *	21.7% (10) *	
*Staphylococcus hominis*	18.1% (32) *	17.6% (23) *	19.7% (9) *	
*Staphylococcus epidermidis*	11.3% (20)	10.7% (14)	13.0% (6)	
*Staphylococcus haemolyticus*	10.2% (18)	9.9% (13)	10.9% (5)	
*Staphylococcus warneri*	2.8% (5)	3.8% (5)	0.0% (0)	0.91 (F)
*Staphylococcus auricularis*	1.1% (2)	1.5% (2)	0.0% (0)	
*Staphylococcus sciuri*	1.1% (2)	1.5% (2)	0.0% (0)	
*Staphylococcus cohnii*	1.1% (2)	0.8% (1)	0.0% (0)	
*Staphylococcus lugdunensis*	0.6% (1)	0.8% (1)	2.2% (1)	
*Staphylococcus simulans*	0.6% (1)	0.8% (1)	0.0% (0)	
*Staphylococcus saprophyticus*	0.6% (1)	0.0% (0)	2.2% (1)	

* Significance test (*p* < 0.05). C = chi square test; F = Fisher’s exact test; MW = Mann–Whitney test; non-ICU = non-intensive care unit; ICU = intensive care unit.

**Table 2:** Distribution of staphylococcal species and antimicrobial resistance in SARS-CoV-2-negative patients.

Antibiotic	*S. aureus* *N* = 46	*S. auricularis* *N* = 2	*S. capitis* *N* = 23	*S. cohnii* *N* = 1	*S. epidermidis* *N* = 14	*S. haemolyticus* *N* = 13	*S. hominis* *N* = 23	*S. lugdunensis* *N* = 1	*S. sciuri* *N* = 2	*S. simulans* *N* = 1	*S. warneri* *N* = 5
Fusidic acid	22.2% (10/45)	0.0% (0/2)	26.1% (6/23)	0.0% (0/1)	50.0% (7/14)	61.5% (8/13)	45.5% (10/22)	0.0% (0/1)	100% (2/2)	0.0% (0/1)	50.0% (2/4)
Penicillin	93.0% * (40/43)	-	-	-	-	-	-	0.0% (0/1)	-	-	-
Clindamycin	42.2% (19/45)	50.0% (1/2)	40.9% (9/22)	0.0% (0/1)	57.1% (8/14)	69.2% (9/13)	77.3% * (17/22)	0.0% (0/1)	50.0% (1/2)	100% (1/1)	50.0% (2/4)
Daptomycin	24.4% (11/45)	0.0% (0/2)	43.5% (10/23)	0.0% (0/1)	50.0% (7/14)	23.1% (3/13)	22.7% (5/22)	0.0% (0/1)	50.0% (1/2)	0.0% (0/1)	0.0% (0/5)
Erythromycin	60.0% * (27/45)	50.0% (1/2)	36.4% (8/22)	100% (1/1)	64.3% (9/14)	100% (13/13)	90.9% * (20/22)	0.0% (0/1)	100% (2/2)	100% (1/1)	80.0% (4/5)
Gentamicin	35.6% (16/45)	0.0% (0/2)	73.9% * (17/23)	0.0% (0/1)	42.9% (6/14)	100% (13/13)	45.5% (10/22)	0.0% (0/1)	50.0% (1/2)	0.0% (0/1)	20.0% (1/5)
Levofloxacin	62.2% * (28/45)	100% (2/2)	69.6% * (16/23)	0.0% (0/1)	78.6% (11/14)	100% (13/13)	56.5% (13/23)	100% (1/1)	100% (2/2)	0.0% (0/1)	60.0% (3/5)
Linezolid	8.9% (4/45)	0.0% (0/2)	13.0% (3/23)	0.0% (0/1)	28.6% (4/14)	23.1% (3/13)	17.4% (4/23)	0.0% (0/1)	0.0% (0/2)	0.0% (0/1)	0.0% (0/5)
Oxacillin	65.2% * (30/46)	50.0% (1/2)	78.3% * (18/23)	100% (1/1)	85.7% * (12/14)	100% (13/13)	76.2% * (16/21)	0.0% (0/1)	100% (2/2)	100% (1/1)	40.0% (2/5)
Rifampicin	40.0% (18/45)	0.0% (0/2)	13.6% (3/22)	0.0% (0/1)	42.9% (6/14)	84.6% (11/13)	45.5% (10/22)	0.0% (0/1)	50.0% (1/2)	0.0% (0/1)	20.0% (1/5)
Teicoplanin	24.4% (11/45)	0.0% (0/2)	17.4% (4/23)	0.0% (0/1)	50.0% (7/14)	37.5% (3/8)	26.1% (6/23)	0.0% (0/1)	0.0% (0/2)	0.0% (0/1)	60.0% (3/5)
Tetracycline	11.1% (5/45)	0.0% (0/2)	8.7% (2/23)	0.0% (0/1)	21.4% (3/14)	38.5% (5/13)	27.3% (6/22)	0.0% (0/1)	0.0% (0/2)	0.0% (0/1)	20.0% (1/5)
Tigecycline	6.7% (3/45)	0.0% (0/2)	4.3% (1/23)	0.0% (0/1)	14.3% (2/14)	15.4% (2/13)	4.5% (1/22)	0.0% (0/1)	0.0% (0/2)	0.0% (0/1)	0.0% (0/5)
Trimethoprim-sulfamethoxazole	8.9% (4/45)	0.0% (0/2)	0.0% (0/23)	0.0% (0/1)	14.3% (2/14)	46.2% (6/13)	4.3% (1/23)	0.0% (0/1)	0.0% (0/2)	0.0% (0/1)	0.0% (0/5)
Vancomycin	17.8% (8/45)	0.0% (0/2)	8.7% (2/23)	0.0% (0/1)	35.7% (5/14)	23.1% (3/13)	13.0% (3/23)	0.0% (0/1)	0.0% (0/2)	0.0% (0/1)	20.0% (1/5)

* Significance test (*p* < 0.05). Cochran’s Q-test with the minimum required differences method and the Bonferroni *p*-value-corrected post hoc test were performed.

**Table 3:** Distribution of staphylococcal species and antimicrobial resistance in SARS-CoV-2-positive patients.

Antibiotic	*S. aureus* *N* = 14	*S. capitis* *N* = 10	*S. epidermidis* *N* = 6	*S. haemolyticus* *N* = 5	*S. hominis* *N* = 9	*S. lugdunesis* *N* = 1	*S. saprophyticus* *N* = 1
Fusidic acid	25% (3/12)	20% (2/10)	50% (3/6)	80% (4/5)	11.1% (1/9)	0.0% (0/1)	100% (1/1)
Penicillin	85.7% * (12/14)	-	-	-	-	0.0% (0/1)	-
Clindamycin	50% (7/14)	40% (4/10)	50% (3/6)	60% (3/5)	22.2% (2/9)	100% (1/1)	100% (1/1)
Daptomycin	14.3% (2/14)	20% (2/10)	0.0% (0/6)	0.0% (0/5)	0.0% (0/9)	0.0% (0/1)	0.0% (0/1)
Erythromycin	57.1% * (8/14)	40% (4/10)	100% * (6/6)	60% (3/5)	44.4% (4/9)	100% (1/1)	100% (1/1)
Gentamicin	0.0% (0/14)	80% * (8/10)	33.3% (2/6)	60% (3/5)	44.4% (4/9)	0.0% (0/1)	100% (1/1)
Levofloxacin	42.9% (6/14)	80% * (8/10)	66.7% (4/6)	60% (3/5)	55.6% * (5/9)	0.0% (0/1)	100% (1/1)
Linezolid	7.1% (1/14)	10% (1/10)	0.0% (0/6)	0.0% (0/5)	0.0% (0/9)	0.0% (0/1)	100% (1/1)
Oxacillin	42.9% (6/14)	90% * (9/10)	100% * (6/6)	40% (2/5)	55.6% * (5/9)	0.0% (0/1)	100% (1/1)
Rifampicin	14.3% (2/14)	40% (4/10)	16.7% (1/6)	20% (1/5)	22.2% (2/9)	0.0% (0/1)	100% (1/1)
Teicoplanin	7.1% (1/14)	30% (3/10)	16.7% (1/6)	20% (1/5)	0.0% (0/9)	0.0% (0/1)	0.0% (0/1)
Tetracycline	14.3% (2/14)	10% (1/10)	16.7% (1/6)	20% (1/5)	0.0% (0/9)	0.0% (0/1)	0.0% (0/1)
Tigecycline	7.1% (1/14)	10% (1/10)	0.0% (0/6)	0.0% (0/5)	0.0% (0/9)	0.0% (0/1)	0.0% (0/1)
Trimethoprim-sulfamethoxazole	7.1% (1/14)	0.0% (0/10)	16.7% (1/6)	0.0% (0/5)	0.0% (0/9)	0.0% (0/1)	0.0% (0/1)
Vancomycin	14.3% (2/14)	10% (1/10)	0.0% (0/6)	0.0% (0/5)	0.0% (0/9)	0.0% (0/1)	0.0% (0/1)

* Significance test (*p* < 0.05). Cochran’s Q-test with the minimum required differences method and the Bonferroni *p*-value-corrected post hoc test were performed.

**Table 4:** Significance results obtained by comparing *Staphylococcus* spp. antimicrobial resistance between SARS-CoV-2-negative and positive groups (Table 2 vs. Table 3).

Antibiotic	*Staphylococcus* spp.	SARS-CoV-2: Negative vs. Positive
Gentamicin	*S. aureus*	35.6 vs. 0.0, *p* = 0.007 * (F)
Oxacillin	*S. haemolyticus*	100 vs. 40.0, *p* = 0.012 * (F)
Rifampicin	*S. haemolyticus*	84.6 vs. 20.0, *p* = 0.022 * (F)
Clindamycin	*S. hominis*	77.3 vs. 22.2, *p* = 0.012 * (F)
Erythromycin	*S. hominis*	90.9 vs. 44.4, *p* = 0.012 * (F)

* Significance test (*p* < 0.05). F = Fisher’s exact test; *p* = *p*-value.

## Data Availability

Not applicable.
